# The Adolescent Girls Initiative-Kenya (AGI-K): study protocol

**DOI:** 10.1186/s12889-016-2888-1

**Published:** 2016-03-01

**Authors:** Karen Austrian, Eunice Muthengi, Joyce Mumah, Erica Soler-Hampejsek, Caroline W. Kabiru, Benta Abuya, John A. Maluccio

**Affiliations:** Population Council, P.O. Box 17643-00500, Nairobi, Kenya; African Population and Health Research Center, P.O. Box 10787-00100, Nairobi, Kenya; Population Council, One Dag Hammarskjold Plaza, New York, NY 10017 USA; Middlebury College, 14 Old Chapel Rd, Middlebury, VT 05753 USA

**Keywords:** Adolescent girls, Randomized trial, Multi-sectoral, Kenya, Schooling, Conditional cash transfer, Savings, Financial education, Violence prevention, Education, Health

## Abstract

**Background:**

Many adolescent girls in Kenya and elsewhere face considerable risks and vulnerabilities that affect their well-being and hinder a safe, healthy, and productive transition into early adulthood. Early adolescence provides a critical window of opportunity to intervene at a time when girls are experiencing many challenges, but before those challenges have resulted in deleterious outcomes that may be irreversible. The Adolescent Girls Initiative-Kenya (AGI-K) is built on these insights and designed to address these risks for young adolescent girls. The long-term goal of AGI-K is to delay childbearing for adolescent girls by improving their well-being.

**Intervention:**

AGI-K comprises nested combinations of different single-sector interventions (violence prevention, education, health, and wealth creation). It will deliver interventions to over 6000 girls between the ages of 11 and 14 years in two marginalized areas of Kenya: 1) Kibera in Nairobi and 2) Wajir County in Northeastern Kenya. The program will use a combination of girl-, household- and community-level interventions. The violence prevention intervention will use community conversations and planning focused on enhancing the value of girls in the community. The educational intervention includes a cash transfer to the household conditioned on school enrollment and attendance. The health intervention is culturally relevant, age-appropriate sexual and reproductive health education delivered in a group setting once a week over the course of 2 years. Lastly, the wealth creation intervention provides savings and financial education, as well as start-up savings.

**Methods/Design:**

A randomized trial will be used to compare the impact of four different packages of interventions, in order to assess if and how intervening in early adolescence improves girls’ lives after four years. The project will be evaluated using data from behavioural surveys conducted before the start of the program (baseline in 2015), at the end of the 2-year intervention (endline in 2017), and 2 years post-intervention (follow-up in 2019). Monitoring data will also be collected to track program attendance and participation. Primary analyses will be on an intent-to-treat basis. Qualitative research including semi-structured interviews of beneficiaries and key adult stakeholders in 2016 and 2018 will supplement and complement the quantitative survey results. In addition, the cost-effectiveness of the interventions will be assessed.

**Discussion:**

AGI-K will provide critical evidence for policy-makers, donors and other stakeholders on the most effective ways to combine interventions for marginalized adolescent girls across sectors, and which packages of interventions are most cost-effective.

**Trial registration:**

ISRCTN77455458, December 24, 2015

**Electronic supplementary material:**

The online version of this article (doi:10.1186/s12889-016-2888-1) contains supplementary material, which is available to authorized users.

## Background

The Adolescent Girls Initiative-Kenya (AGI-K) is a randomized trial designed to test whether different combinations of 2-year long multi-sectoral interventions, targeted to young adolescent girls (11–14 years) can improve their well-being after 4 years (when aged 15–18 years), and in doing so enable safe, healthy and productive transitions into young adulthood. In this paper, we describe the rationale, program design and research methods underlying AGI-K.

Many adolescent girls in Kenya, and other countries in sub-Saharan Africa, face considerable risks and vulnerabilities that affect their health and general well-being. These risks and vulnerabilities include low educational attainment and illiteracy, household poverty, lack of economic independence, limited income earning opportunities, exposure to violence, and social isolation [1]. For the most part, adolescent girls younger than 15 year who live in environments laden with these risks and vulnerabilities have not yet experienced the associated critical negative outcomes including; school dropout, first sex, unintended pregnancy, early marriage, and experience of sexual and gender based violence. Thus, early adolescence provides a critical window of opportunity to intervene at a time when girls are experiencing many challenges, but before those challenges have resulted in outcomes that may be irreversible [2].

A wide range of research has shown the multiple benefits of educating girls, including improved reproductive health [3–6]. Documented benefits include delaying first marriage, lowering family size, improved health for them and their children, as well as economic benefits to a woman, her family, and community. However, new thinking on the effects of education on well-being also posits that education alone is not enough to achieve levels of ‘empowerment,’ needed for successful transitions into adulthood, and that girls need critical thinking skills as well as an enabling environment such as family/community and societal commitment to and capacity for educating girls [7].

Evidence also suggests that economic assets, in addition to having value on their own, have benefits in other areas of women and girls’ lives. For example, girls who have fewer economic assets are more likely to have exchanged sex for money, gifts or shelter compared to girls with more assets [8]. Studies have also shown that girls who received a cash benefit for schooling were less likely to marry early and to report sexual activity and teenage pregnancy [9]. Further, there is evidence that adding a financial education component to a life-skills intervention resulted in significantly greater positive impact in changing sexual behaviours [10]. A lack of economic assets has also been cited as a barrier to translating sexual and reproductive health knowledge into behaviour change, especially during adolescence, as girls are often financially dependent on men and therefore lose decision making power in their sexual relationships [11]. However, economic interventions on their own may not be sufficient to achieve desired outcomes, and can even increase risk among adolescents [12, 13], while programmes that have combined economic strengthening interventions with prevention of violence and health components have had a positive outcome on all three areas [14].

**Fig. 1:**
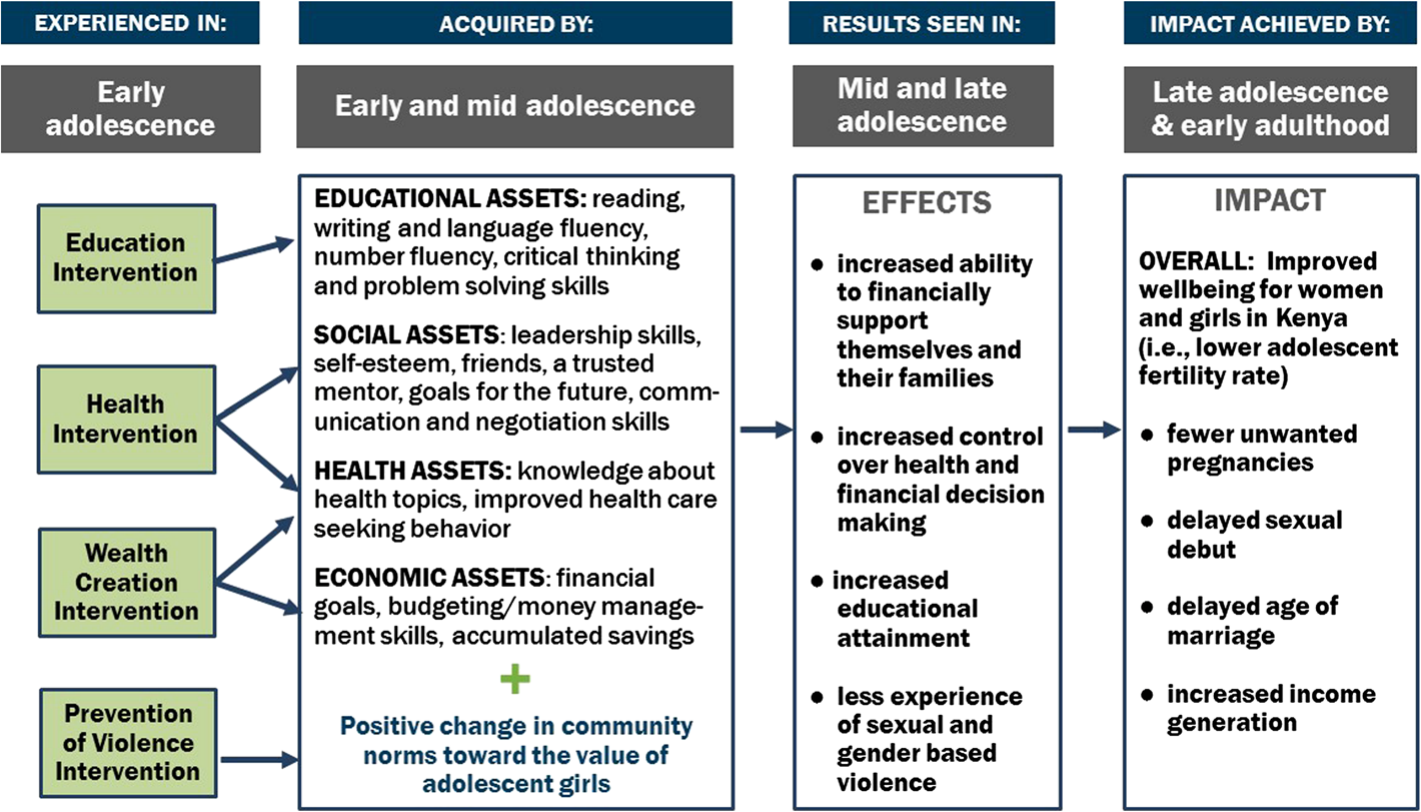
AGI-K theory of change

Collectively, the evidence points to multi-sectoral approaches for adolescent girls programming as a promising strategy for achieving high levels of impact. The interventions for AGI-K were therefore based on the Asset Building Theory of Change that posits that girls need a combination of education, social, health, and economic assets to make a safe, healthy, and productive transition from adolescence into young adulthood [15, 16]. In addition, community norms regarding girls’ values must be strengthened to facilitate the increase in assets for girls and the resulting improvements in medium- and longer-term outcomes (see Fig. 1). The hypothesis is that this diverse asset base, once acquired, will lead to increased educational attainment, delayed marriage and childbearing, fewer unintended pregnancies, less experience of violence, and increased income generation.

AGI-K is being implemented in two different marginalized sites in Kenya. One is Kibera, a densely populated urban informal settlement in the capital city of Nairobi. Kibera is characterized by an ethnically and religiously diverse population, high levels of household poverty and crime, and lack of formal basic services [17]. The other is rural Wajir County, a sparsely populated rural area on the Kenya-Somali border that is made of an ethnically (Somali) and religiously (Muslim) homogenous population. This county has some of the poorest health, education and socio-economic indicators in the country [1, 18].

## Program design

The long-term goal of AGI-K is to delay childbearing for adolescent girls by improving their well-being. Based on the Asset Building Theory of Change, AGI-K will implement intervention packages comprised of nested combinations of “sector-specific” interventions in order to estimate the incremental effect of each additional intervention, as well as their combined effects. Below we describe the four sector-specific interventions—violence prevention, education, health, and wealth creation—each of which lasts for 2 years. The interventions are being implemented by non-governmental organizations, referred to here as the implementing partners^1^.

### Violence prevention

For AGI-K, the definition of violence against girls and women in the community includes sexual and physical violence, but also incorporates a broader conceptualization of violence as the de-valuation of girls, for example including female genital mutilation/cutting (FGM/C), lack of education and early marriage [19, 20]. To prevent violence, therefore, the program will implement a community-level intervention based on the Concern International/United Nations Development Programme (UNDP) Community Conversations (CC) model [21]. Although not rigorously evaluated, the CC model has several features demonstrated in other contexts to be important to the success of similar community-level interventions [14, 20, 22, 23]—it engages in dialogue a variety of community members (including key stakeholders who are often the gatekeepers to girls’ well-being) who are charged with: 1) identifying problems including, for example, inhibiting gender norms or attitudes; 2) designing and implementing their own context-specific solutions; and, in doing so 3) developing a sense of ownership in addressing social issues [24].

The first step in implementing the CC model is to establish in each village a core committee comprising religious and community leaders, parents, teachers, and young men and women. The committee members participate in facilitated discussions (which follow a prescribed schedule with specific session topics, activities and tools) in which they identify the key drivers of de-valuation of girls in the community, develop a written “contract” including specific steps to address those determinants, and then carry out activities to implement the contract over the course of the 2-year intervention period. Up to 100,000 Kenyan Shillings (KES) (approximately 1000 US dollars^2^) per community are available to each community to aid in fulfilling the contract, provided as in-kind materials or services by the implementing partner. Committee meetings (and attendance at each) are monitored and recorded by the program in an electronic administrative database, as are all payment amounts for the in-kind transfers and services provided. Progress on, and completion of, the contracted activities are monitored by the implementing partner.

### Education

The education sector intervention will comprise cash and in-kind transfers conditioned on school enrollment and attendance in either primary or secondary school. Randomized studies show that similar transfers have been successful in improving schooling outcomes elsewhere in sub-Saharan Africa [25–27]. A global systematic review of such conditional cash transfer (CCT) programs concluded that CCTs are an effective intervention for increasing school attendance, and when closely monitored are more effective than unconditional transfers (UCT) [28]. Experimental results from a different UCT program in Kenya targeting orphans and vulnerable children also does not find an overall average treatment effect on most schooling outcomes, although it points to possible heterogeneous effects for important subgroups including those facing higher school costs and those transitioning to secondary school as expected for many of the girls in the AGI-K intervention in Kibera [29]. In Western Kenya, small education subsidies (in the form of free uniforms for up to two school years) provided to girls enrolled in sixth grade not only reduced dropout in the short-term but also increased grades completed measured 7 years after the start of the intervention [30].

Girls are eligible for the AGI-K CCT regardless of initial schooling status at the start of the program (i.e., whether or not enrolled in school at baseline). Kenyan schools operate on the calendar year with three distinct 3-month terms each year so that the 2-year program covers six school terms. Transfers are made for any school term during the intervention period in which the girl meets all relevant enrollment and attendance conditions. School enrollment is verified using school records while school attendance is monitored using biometric fingerprint reading at the start and end of each school day, triangulated with school-level attendance register records.

There are four components to the education transfer:

1. *Household Cash Transfer:* The household head (or other designated household member) receives two cash transfers per term, transferred into a bank account. In Kibera, the amount is 1125 KES per transfer, for a total of 2250 KES per term. In Wajir, the amount is 1250 KES per transfer, for a total of 2500 KES per term. The total transfer per term was calculated to reflect 10 % of average household expenditures for a 4 month period. The amount was benchmarked to similar education cash transfer programs [27]. For the first term covered by the intervention (the second term of the 2015 school year) and the first term of each new school year, the first of the two transfers per term is conditional only on enrollment. For all other terms, the first of the two transfers per term is conditional on at least 80 % attendance during the first month of that term. For all six covered school terms, the second transfer per term is conditional on at least 80 % attendance during the second and third months, and is made after the third month.
2. *School Fees:* If applicable, school fees are paid directly to the school at the start of each school term, up to a maximum of 1200 KES per term for primary school and 6000 KES per term for secondary school in Kibera, and 700 KES and 5000 KES, respectively, in Wajir. Maximum transfers were based on the mean fee amounts parents reported paying out of pocket during the needs assessments in each area. For the first term covered by the intervention, as well as the first term of each new school year, school fee payments are conditioned on enrollment (the same conditionality as for the first household cash transfer in each of those terms). Payment of second and third term fees is conditioned on at least 80 % attendance during the whole of the prior term, and continued enrollment.
3. *School Incentive:* Once each term, schools also receive an additional 500 KES per girl enrolled in the CCT program and paid at the same time as the school fees.
4. *Schooling Kits:* At the start of each term, each girl receives a schooling kit with the following items: four packs of sanitary towels, two pairs of underwear, a small container of petroleum jelly, a bar of soap, an exercise book and a pen. The eligibility for the provision of the kit follows the same conditionality as the school fees.

Transfers, including the schooling kit, are only made upon verification that the relevant conditions have been fulfilled. All transfers made as part of the interventions are recorded in the administrative database, which includes all AGI-K girls and the schools they are attending. Biometric fingerprint attendance data are also recorded.

### Health

The health sector intervention focuses on education and will follow the Population Council’s Safe Spaces model in which girls meet in the same groups once a week under the guidance of an adult female mentor from the community [31]. Given the young ages of the girls, the program prioritized health education over access to health services for the intervention in this sector. There is growing evidence that the Safe Spaces model has been effective in improving various social and health-related outcomes in urban Kenya and elsewhere in sub-Saharan Africa [32, 33]. AGI-K uses an adult mentor, rather than a peer-educator of the same age, because the latter has been shown to have limited impacts, with the possible exception of positive effects for the peer educators themselves [34, 35].

AGI-K Safe Spaces groups are segmented by age (11–12 vs. 13–14 years) in Kibera, and by age and/or schooling status in Wajir (11–12 vs. 13–14 years; in-school vs. out-of-school). The groups follow a structured curriculum, but importantly allow substantial time for open discussion. The curriculum was adapted from existing modules used in similar interventions, but modified by the research team and implementing partners for each age group and geographical setting (Kibera versus Wajir) to ensure it was culturally appropriate and acceptable to the communities. It includes material on hygiene, nutrition, HIV/AIDS, sexual and reproductive health, communication and negotiation skills, gender norms, sexual and gender-based violence, early marriage, leadership skills and relationships.

Groups meet at locations in the community identified as safe and appropriate for the young girls, including community halls, schools, churches, mosques or community leaders’ residences. The groups meet weekly over the course of 2 years. This intervention length is similar to other AGI-K interventions, as well as to other programs using the model. While in part necessary to deliver and reinforce all of content, the prolonged period with repeated interactions also serves to help girls build social assets such as strong friendships and relationships with their adult mentors. Attendance of girls at group meetings is monitored and recorded in the administrative data; mentors contact girls who are absent, including making home visits for girls who are repeatedly absent.

### Wealth creation

The wealth creation intervention is composed of savings and financial education, and savings-targeted transfers. The financial education curriculum is delivered in the Safe Spaces group sessions described above. It is designed to help girls develop basic money-management skills such as budgeting, saving and differentiating between needs and wants, so that they can build economic assets for themselves. Non-experimental evidence indicates that savings and financial education can be effective methods of economic strengthening for young adolescent girls [10, 13].

Two recent reviews of financial education make clear that it is most effective in promoting sustained behaviour change if the lessons can be put into practice at the same time as the education [36, 37]. The design of the AGI-K wealth creation component, therefore, includes an immediate opportunity for girls to begin implementing their budgets and working toward their savings goals. In Kibera, girls are helped to open a SMATA Youth Account with the Kenya Post Office Savings Bank (Postbank) after the first unit of the financial education sessions. These accounts are managed by the girls themselves, although they must select an adult (age 18 or over) as a co-signatory to open the account and to make withdrawals. The accounts have a 200 KES minimum operating balance; the program covers this amount and then makes an additional 300 KES deposit at account opening, for a total of 500 KES. In Wajir, girls receive a home bank (piggy bank) with 300 KES in cash deposited in it after completing the first unit of the financial education sessions. For girls in both sites, an additional 300 KES will be deposited at the start of the second year of the intervention. As with all other transfers, the savings-targeted transfers are recorded in the administrative database.

## Methods/Design

The study is a randomized trial designed to test the relative effectiveness of the following nested combinations of the sector-specific interventions described above:

1. Violence Prevention
2. Violence Prevention + Education
3. Violence Prevention + Education + Health
4. Violence Prevention + Education + Health + Wealth Creation

Conceptual, as well as logistical considerations determined the selection of the specific combinations of interventions to be compared across the four arms of the experiment. As a community-wide intervention, violence prevention was considered non-excludable in the geographically small and densely populated urban informal settlements of Kibera, characterized by high internal mobility [17]. In Wajir, on the other hand, community leaders indicated it would not be socially acceptable to conduct research without providing some type of direct benefit to all communities in the research sample. Therefore, even though excludability was theoretically possible at the community level, randomized control communities were deemed infeasible in the context of Wajir. For these reasons, we implement the community-wide violence prevention intervention across all randomized units in both sites. An advantage to doing this is that it directly addresses the enabling environment for all girls, for example addressing social norms regarding the value for girls in the communities, and engaging the community in ensuring that girls are safe from all forms of violence in their homes, schools and communities (Fig. 1).

The AGI-K consortium next added education, as this was hypothesized to have the greatest impact in reducing fertility and improving the wellbeing of girls in the future, based on the existing evidence [3–6]. The health intervention using the Safe Spaces model, with its more direct emphasis on improving reproductive health outcomes, was added next. And, last, wealth creation was incorporated into the fourth arm, which includes all four intervention sectors. This layering allows direct comparison of packages with and without Safe Spaces (V & V + E versus V + E + H & V + E + H + W) as well as between packages where Safe Spaces includes health only (V + E + H) versus combined health and wealth creation (V + E + H + W). By comparing each arm with the next, we are able to test the additional benefit of adding each of the three interventions (education, health and wealth) to the previous arm. In addition, comparing the third and fourth arms to the first allows one to test the additional benefit of adding wealth creation to the combination of education and health. While there is growing evidence that combined health and wealth creation are important [10, 13, 38], this study examines the impact of layering this combination onto violence prevention and education.

Finally, the cost of implementing each package will be different, increasing with the number of sectors. Sequentially adding interventions so that they are nested puts the cost-effectiveness assessment on a much stronger basis (by avoiding comparisons across multi-sectoral interventions with mutually exclusive sets of single-sector interventions that target different outcomes).

The intervention packages will be evaluated using data from behavioural surveys conducted before the start of the program (baseline in 2015), at the end of the 2-year intervention (endline in 2017), and two years post-intervention (follow-up in 2019). The survey data will be complemented by administrative data on schooling and Safe Spaces groups attendance as well as transfers collected for monitoring purposes throughout program operation. A complementary cost-effectiveness study also will be carried out using program administrative data on costs. Qualitative research including semi-structured interviews of beneficiaries and key adult stakeholders in 2016 and 2018 will supplement and complement the quantitative survey results. The qualitative research will include assessments of the fidelity of program implementation and exploration of the mechanisms underlying estimated impact of the interventions.

The unit of randomization is different for the two sites: individual-level randomization in Kibera and cluster randomization in Wajir. In densely populated Kibera, an individual-level randomized design was logistically possible with excludable interventions (with violence prevention included in all arms) and preferable given that for any fixed number of girls it yields greater statistical power than a cluster design. A nearby non-experimental external control site also was included in the design to enable comparison of girls in each of the experimental arms with girls in a similar area that will not receive any of the four program interventions. In particular, this allows a non-experimental assessment of the impact of the violence prevention only intervention. In Wajir, which is less densely populated and characterized by distinct and non-adjacent settlements, individual randomization was not feasible and instead a cluster-level design followed, without any pure control.

### Study outcomes

The overall objective of AGI-K is to improve the well-being of beneficiary girls after four years (when aged 15–18 years), delaying childbearing and enabling safe, healthy and productive transitions into young adulthood. Consequently, while we specify a number of outcomes (and associated indicators) related to this comprehensive objective, there is no single outcome indicator that fully captures it; rather, evidence must be drawn from a combination of relevant outcome indicators. In Table 1, we organize the most important of these into primary and secondary outcomes, and their related indicators. The primary outcome indicators for the study are linked to delayed childbearing and include age at first birth, age at first sex and age at first marriage. Secondary outcome indicators span the four domains directly targeted by the sector-specific interventions.

**Table 1:** Key indicators for primary and secondary outcomes

Outcome domain	Indicator 1	Indicator 2	Indicator 3
Primary outcome			
Well-being	Age at first birth (+)	Age at first sex (+)	Age at marriage (+)
Secondary outcomes			
Violence	Experience of gender-based violence (−)	Positive gender norms related to violence (+)	
Education	Mean grade of schooling (+)	Rate of primary school completion (+)	
Health	Knowledge on sexual and reproductive health (+)	Decision-making skills (+)	Contraceptive use (+)
Wealth	Knowledge on financial education (+)	Saving (+)	Participation in income generating activities (+)

### Hypotheses

**Fig. 2:**
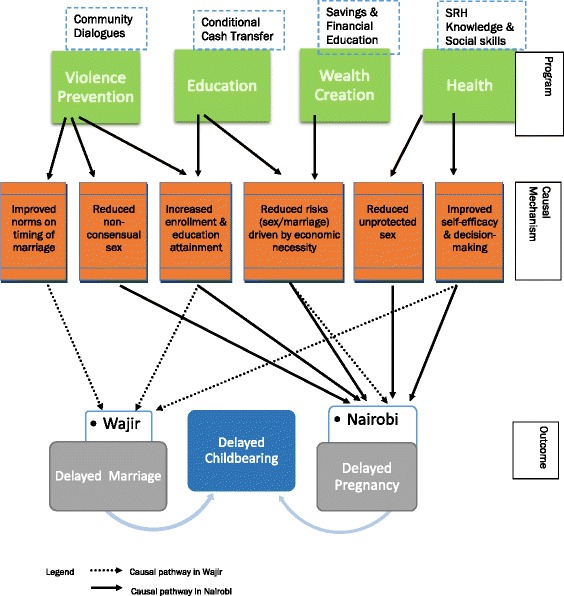
AGI-K causal mechanisms

The study will test several hypotheses, as well as explore the underlying causal mechanisms shown in Fig. 2. The long-term goal of the AGI-K interventions is to delay childbearing. Each proposed intervention is hypothesized to delay childbearing through different causal mechanisms. In Kibera, we hypothesize that the primary pathways to delaying childbearing are delaying sexual debut and/or increasing contraceptive use. In Wajir, on the other hand, we hypothesize that the primary pathway is delaying marriage. Below is a description of the intervention specific hypotheses.

#### Violence prevention

The violence prevention intervention is hypothesized to have a positive effect on the value of girls at the community level. In Kibera, this increased value is expected to change attitudes, perceptions and norms around sexual violence against girls, leading to a decrease in unintended pregnancies by reducing the incidence of non-consensual sex. In Wajir, the increased value of girls is expected to change attitudes, perceptions and norms on the timing of marriage for girls (e.g., that it is better for girls to marry when older). This change in attitudes, perceptions and norms is expected to delay marriage directly, which in turn is expected to delay childbearing, since in Wajir virtually all births occur within marriage [1]. In both Kibera and Wajir, the intervention is also expected to increase the importance that the community places on educating girls, in general, which would lead to increased schooling. Increased educational attainment is further expected to delay marriage and childbearing [4, 39].

#### Education

The education CCT intervention is hypothesized to delay childbearing through two main mechanisms. The first is through girls staying in school longer [39–42]. In Kibera, increased schooling is expected to delay childbearing directly, while in Wajir it is expected to influence childbearing through delaying marriage. The second mechanism is through increased household income from the cash transfers, which is hypothesized to reduce risks to girls associated with lack of economic resources at the household level. In Wajir, it is hypothesized that the cash transfers will offset the economic incentive for parents to marry off their daughters, thus delaying marriage.

#### Health

The health education intervention is hypothesized to lead to increased knowledge of sexual and reproductive health, as well as increased social support through strengthened peer networks and relationships with adult female mentors. This intervention is expected to delay childbearing via two key mechanisms. The first mechanism is through increased sexual and reproductive health knowledge, which will primarily be relevant in the Kibera setting, and which is expected to lead to a reduction in unprotected sex—either by delaying sexual debut or increasing contraceptive use. The second is through improved self-efficacy. In Kibera, improved self-efficacy is expected to enable girls to improve their sexual behaviours—either by choosing to delay sex or through increased ability to negotiate contraceptive use during sex. Improved self-efficacy in Wajir is expected to be reflected in girls having a greater say in the timing of their marriage, or being able to engage community leaders (e.g., through their mentor) to intervene to stop or delay a planned marriage.

#### Wealth creation

The wealth creation intervention is hypothesized to improve financial literacy and the accumulation of savings. In Kibera, it is hypothesized that greater access to economic assets, and financial independence associated with savings, will allow girls to have more control over their sexual partnerships, for example decreasing the likelihood of engaging in transactional sex which is often unsafe [11]. Therefore, a decrease in these types of sexual relationships is expected to reduce unintended pregnancies and delay childbearing. In both settings, though relatively more important in Wajir where early marriage is common, greater access to economic assets and financial independence, is expected to improve girls’ role in decision-making. Ultimately, increased decision-making skills are expected to lead to girls having increased control over health decisions, including the use of contraceptives to delay first birth.

### Study sites and sample sizes

Study sites were selected to investigate similar comprehensive interventions in two marginalized, but very different, contexts in Kenya: 1) urban informal settlements in Nairobi and 2) rural village settlements in semi-arid Northeastern Kenya. Due to the differences in context (including for example population density and available education and health services), cultures and local economies and prices (influencing costs), the study consists of two sub-studies-the Nairobi and Wajir sub-studies—with an independent dataset for each site. In each site, the research is confined to specific geographical areas selected in part based on analysis of the Kenya National Bureau of Statistics (KNBS) 2009 National Census, which was used to predict population sizes for the target girls, as well as on other considerations specific to each site (and described below). Consequently, administrative units are defined as in that census using the pre-devolution^3^ units of Kenyan Districts, Divisions, Locations, and Sub-Locations.

Limited evidence from similar contexts on potential effect sizes of these comprehensive interventions led to an indirect approach to sample size calculations, first estimating the maximum sample size possible given the research and intervention budget for the study and then turning to the limited literature to explore whether such effect sizes were plausible. For reporting purposes, then, we focus on the minimum detectable effect sizes permitted by the maximum and eventual attained samples.

The budget provided sufficient funds for a maximum sample size of approximately 3000 girls in each study site. Differences in context led to the more powerful individual randomization research design in Kibera and a randomized cluster design in Wajir. Based on an initial target sample of 3000 girls in Kibera, and 80 clusters with 40 girls each (for a sample of 3200 girls) in Wajir, we estimated minimum detectable effects assuming 20 % loss to follow-up for two outcomes for which we had reliable data (detailed below). Updated information after carrying out program-related censuses in the selected areas revealed that the maximum research sample size was unattainable, however. For each site, therefore, we recalculate the minimum detectable effects for the smaller, attained sample, and compare the differences.

The primary target population includes all girls between 11 and 14^4^ years who were residing within selected study sites and who were not enrolled in boarding school (and therefore living away from home during school terms) at the time of the household listing and at the time of the baseline survey^5^. Parents, guardians, and community members residing there are also targeted within the violence prevention intervention. Minimum detectable effects on percent of girls who have given birth (a primary outcome) and grades attained (a secondary outcome) for the maximum possible samples were estimated.

Data from the 2012 Nairobi Cross-Sectional Slum Survey (NCSSS) were used to obtain estimates of baseline childbearing and education used for the power calculations using individual randomization in Kibera [17]. The maximum feasible sample size was 750 girls per arm at baseline (600 girls per arm at follow-up, assuming a loss of 20 %). However, due to a higher than expected proportion of non-eligible girls (i.e., enrolled in boarding school or having moved out of the study area prior to the program start), the attained sample included approximately 600 girls per arm at baseline (480 girls per arm at follow up, assuming a loss of 20 %).

Data for the Northeastern Province from the 2008/09 Kenya Demographic Survey were used to obtain estimates of baseline childbearing and education and the intra-cluster correlation (ICC), used for the power calculations using cluster randomization in Wajir [18]. The maximum feasible sample size was 20 clusters per arm and 40 girls per cluster at baseline (32 girls per cluster at follow-up, assuming a loss of 20 %). However, due to differences between population estimates and the actual number of eligible girls residing in these communities at the time of the survey, the final sample included 20 clusters per arm with an average of 27 girls per cluster at baseline (22 girls per cluster at follow-up, assuming a loss of 20 %).

Table 2 shows the minimum detectable differences in each site for the two outcomes and for the maximum and attained sample sizes, assuming attrition rates of 20 % at follow-up, with power of 0.80 and a significance level of 0.05^6^. In both sites, the minimum detectable effects on the percent of girls who have given birth by 2019 is 1 percentage point (approximately 17 %) higher for the attained versus the maximum sample sizes; for grades of schooling, however, the differences are smaller (approximately 10 %).

**Table 2:** Minimum detectable differences for sample estimates

Site	Sample estimate	Minimum detectable differences
Kibera	600 girls per arm at follow-up (2019)	Percent of girls who have given birth: Assuming that 15.4 % of girls in the violence prevention only arm would have given birth by follow-up, can detect a statistically significant difference of 5.4 percentage points between the violence prevention only arm and each of the other three arms
		Grades of schooling: Assuming a correlation coefficient of 0.33, can detect a statistically significant difference of 0.49 grades of schooling between any two arms
	480 girls per arm at follow-up (2019)	Assuming that 15.4 % of girls in the violence prevention only arm would have given birth by follow-up, can detect a statistically significant difference of 6.3 percentage points between the violence prevention only arm and each of the other three arms
		Assuming a correlation coefficient of 0.33, can detect a statistically significant difference of 0.55 grades of schooling between any two arms
Wajir	20 clusters per arm, 32 girls at follow-up (2019)	Assuming that 17.6 % of girls in the violence prevention only arm would have given birth by follow-up, can detect a statistically significant difference of 5.9 percentage points between the violence prevention only arm and each of the other three arms
		Assuming a correlation coefficient of 0.26, can detect a statistically significant difference of 0.48 grades of schooling between any two arms
	20 clusters per arm, 22 girls at follow-up (2019)	Assuming that 17.6 % of girls in the violence prevention only arm would have given birth by follow-up, can detect a statistically significant difference of 6.9 percentage points between the violence prevention only arm and each of the other three arms
		Assuming a correlation coefficient of 0.26, can detect a statistically significant difference of 0.49 grades of schooling between any two arms

These effect sizes are considered attainable for the attained sample, considering the multi-sectoral approach that addresses several of the factors that increase risk of early childbearing in these young populations in which very few girls have begun childbearing. Two large cash transfer programs in Kenya and Malawi using a randomized control trial design obtained similar results using a single-sector approach, albeit for older samples. The Kenya Cash Transfer for Orphans and Vulnerable Children showed a reduced likelihood of pregnancy by 5 percentage points at the Year Four follow-up survey among women aged 12–24 [43]. The Zomba Cash Transfer Program in Malawi, a randomized CCT targeting young women aged 13–24, showed a reduction of 5.1 percentage points in the likelihood of becoming pregnant over 1 year for girls who were out of school at baseline [26]. Nevertheless, to reduce the impact on statistical power of the smaller attained samples, program participants will be monitored at least weekly, and a tracking tool developed and administered to research participants semi-annually to identify their current location and any major transitions, such as school drop-out, marriage or childbirth, in an effort to minimize attrition rates between survey waves.

### Study sites

#### Nairobi sub-study

In Nairobi, all informal settlement sub-locations were identified using population data from the KNBS 2009 national census for Nairobi County. These included: (1) all sub-locations with enumeration areas designated as informal settlements by KNBS in the 2009 National census, as well as (2) all sub-locations with a population density greater than 20,000 people per square kilometre in 2009. The latter were included to ensure coverage of other densely populated areas that were not categorized as informal settlements in 2009. The informal settlement sub-locations were then categorized using KNBS designations of Locations, which are then categorized into Divisions. Areas where significant adolescent girls interventions were already being implemented by the study investigators (Viwandani, Korogocho, and Kariobangi locations), or funded by the same donor (Embakasi division), were excluded.

After excluding high socioeconomic-status areas and areas that did not have a sufficient number of adolescent girls to reach the target (based on 2009 Census data), Kibera was identified as the primary research site. In Kibera Division, seven locations were classified as Urban Slums: Kibera, Lindi, Makina, Silanga, Laini Saba, Soweto/Highrise, Gatwikira, and Olympic. Central division (Huruma sub-location in Huruma location and Mlango Kubwa sublocation in Mathare location) was selected as the external control site due to similarity with Kibera on key characteristics such as parental education, adult employment status and religion [17].

In Nairobi, a complete household listing, to identify eligible girls between the ages of 11 and 14, was conducted in both Kibera and the external control Huruma/Mathare, using maps obtained from KNBS and with the assistance of local leaders. The listing was conducted using Open Data Kit (ODK) software on Android tablets. Enumerators assigned a unique serial number to each household, and if an adult was available they administered a brief screening questionnaire to ascertain whether there was an eligible girl residing within the household. The preferred respondent was the household head, but if unavailable, the second choice was a spouse of the household head, and the third choice was another consenting adult (age 18 or over) residing in the household. Similar to the Demographic and Health Surveys (DHS), a household was defined as one that shares a kitchen (pot) and has the same household head. Household members were defined as individuals who have lived or intend to live in the household for 6 or more months, including school children regularly in residence during the school year (even if they spend time away during school holidays).

A screener was conducted to identify girls in the target population. It had five questions about household demographics but only one (How many girls age 10–15 live in this household?) was used to determine whether the household would be asked further questions. The wider than necessary age range (10–15) was used to ensure eligible girls were not missed due to misreported ages. All households that answered one or more to this question continued to complete a household roster and a brief household survey. The roster asked about all household members and included questions on age, birth year (for adolescents), education, marital status, parents’ survivorship, and number of living children. The household survey included questions on ownership of household assets, source of drinking water, type of toilet facility, number of rooms for sleeping, and the main material of the floor and the roof of the structure.

A total of 64,946 unique households were listed in Kibera and 18,578 households in Huruma/Mathare. The screener was completed with a consenting adult in two-fifths (40 % Kibera; 43 % Huruma/Mathare) of these households, with less than 1 % refusals. Although not completed in the remaining households, the field protocol was designed to ensure age-eligible girls were not missed. In particular, a research assistant visited each household three times and if no one was available inquired from neighbours as to whether there were children between the ages of 10 and 15 years residing in the household. Households with children in that age range were revisited before the research team proceeded to a different enumeration area (or later) such that the number of uninterviewed households with age-appropriate children is likely to be minimal. In Kibera, the listing resulted in 5134 households with at least one girl between the ages of 10 and 15 years, and 4351 girls between the ages of 11 and 14 years. In Huruma/Mathare, the listing resulted in 1348 girls between the ages of 10 and 15 and 1166 girls between the ages of 11 and 14 years.

In Kibera, a total of 4351 girls were determined to be between the ages of 11 and 14 at the time of the listing (based on reported or calculated age). Of these, 611 girls were ineligible because they were immediately identified as being in boarding school (and thus not resident during school term) or residing outside of Kibera for school or other reasons. They were excluded because the aim was to obtain a sample of eligible girls according to the program criteria (i.e., girls who were resident and therefore would be available to participate directly in the program interventions). Of the remaining 3740 girls, one girl per household was randomly selected for the sample, resulting in a sample of 3296 girls in distinct households with 444 siblings (or other girls in the same household) within the target age range. In Huruma/Mathare, a total of 1166 girls were between the ages of 11 and 14, and 153 girls were residing in boarding school or outside of the study area. Of the remaining 1013 girls, one girl per household was randomly selected for the sample, resulting in a sample of 895 girls in distinct households and 118 siblings (or other girls in the same household).

In Kibera, out of the target sample of one randomly selected girl from 3296 distinct households approximately 21 % were confirmed to be ineligible during baseline data collection and could not be successfully replaced by another eligible girl in their same household, based on corrections to their age, enrollment in boarding school (which was not asked during the listing) or no longer being in residence in the community. Of the remaining 2606 eligible girls, 2402 (92 %) were interviewed. In Huruma/Mathare, of the eligible 895 girls from distinct households approximately 18 % were confirmed to be ineligible during baseline data collection and could not be successfully replaced by another eligible girl in the same household, based on age, residence in the community, and being in boarding school. Of the remaining 730 girls, approximately 91 % or 666 were interviewed. The reasons for nonresponse in both Kibera and Huruma/Mathare included refusals by the parent, spouse, or girl herself, incapacitation, death, or inability to locate the household or the girl. The research sample included some girls who were age 14 at the time of the listing but had turned 15 by the time they were interviewed. see Table 3.

**Table 3:** Respondents interviewed

Site	Initial sample from household listing	Eligible	Interviewed (% of eligible)
Kibera	3296	2606 (79 %)	2402 (92 %)
Huruma/Mathare	895	730 (82 %)	666 (91 %)
Wajir	2923	2297 (79 %)	2150 (93 %)

Randomization of individuals to study arms was conducted after the baseline survey in the form of a public lottery for transparency and to minimize confusion and distrust regarding the selection process. Girls were randomly assigned to study arms during a Kibera External Advisory Committee^7^ meeting attended by local stakeholders and leaders. An Excel file with a list of girls’ anonymous ID numbers was projected on the screen, and an Excel formula used to generate a random number for each girl. The list was then sorted in ascending order of the random number and divided into four equally-sized groups based. Four stakeholders volunteered to randomly pick from a bag one of four pieces of paper, each with one of the four study arms written on it, and this arm was assigned to the particular group.

#### Wajir sub-study

In Wajir, clusters were defined as settlements with one public primary school or the primary school-catchment area in settlements with more than one. This ensured that girls had access to a primary school and that they had access to group meeting locations. A total of 80 clusters were identified in Wajir and stratified by district: Wajir West (20 clusters), Wajir East (28 clusters), and Wajir South (32 clusters). Wajir North was excluded because the implementing partner does not operate in that area and it was desirable for the study to work with a single implementing partner in each site. Within the selected three districts, approximately 20 communities were excluded including very small villages with fewer than 8 age-eligible girls, urban areas, peri-urban areas with more than one primary school, and villages on the Kenya-Somali border or other villages where the implementing partner had limited access due to security reasons.

In Wajir, a paper-based listing procedure was used to identify eligible girls in the field, as it was not necessary to randomly select girls for program participation due to the cluster-randomization design. Enumerators visited each household within selected villages, assigned it a unique serial number, and conducted a brief screening interview to obtain the name of the head of the household as well as the number of boys and, separately, girls aged 10–15 years residing in the household. The same definitions for a household and household member were used as described above, as well as the same protocol for the respondent. For households with one or more girls between the ages of 10 and 15, a cover sheet was completed in which enumerators listed the name, age, and sex of each girl and boy in that age group. After listing the entire village, the team leaders collected the cover sheets and counted the number of sheets with a girl in the target age range of 11–14.

A total of 4152 eligible girls were identified in 80 villages, ranging from 9 girls to 181 girls per village. Of these, 2923 were selected for the baseline survey. Selection was determined by the number of eligible girls (ages 11–14 years) in each cluster. For villages with fewer than 40 households with an eligible girl, all households were selected for the baseline sample, and all eligible girls within those households interviewed. In villages with 40 or more households with an eligible girl, team leaders used pre-determined lists of random numbers to randomly select 40 households, and then to randomly select one girl within each household for the baseline survey sample. If that girl turned out to be ineligible and there was another eligible girl in the household, the latter was interviewed.

Of the 2923 girls selected for an interview approximately 21 % were confirmed to be ineligible during baseline data collection, mainly based on residence in the community at the time of the survey or incorrect ages. The survey occurred a few days after the listing, and at that time, these parents and guardians clarified that they had listed girls who no longer resided in the household, or who had migrated away from that community. Of the eligible 2297 girls, 2150 (93 %) were interviewed. The reasons for nonresponse included refusals, incapacitation, death, and inability to locate the girl. see Table 3.

Randomization of clusters to study arms was conducted after the baseline survey, at the district level. In each district, a public meeting was held with stakeholders and local leaders, as well as one representative from each of the clusters. A list of all clusters in the district was displayed on the wall, and a container prepared with the same number of cards as clusters in that district and the cards equally divided among the four study arms, i.e., ¼ of the cards indicating arm 1, ¼ arm 2, etc. A representative from each village selected one card from the container publicly announced the arm selected, and pasted it on the wall next to the name of the cluster. After all the clusters had selected an arm, each representative signed an affidavit acknowledging acceptance of the public lottery results.

### Research instruments

A comprehensive survey at both the household and individual-girl levels was administered prior to program implementation at baseline (2015) (see Additional file 1), and similar surveys will be administered after two years at endline (2017) and after four years at follow-up (2019). In both Nairobi and Wajir, a short household survey was conducted with the head of household or adult providing consent at the time of the interview, collecting information about the household’s experience of major shocks, receipt of cash transfers, participation in other programs and gender norms. In Wajir, this component of the survey also included a household roster and other household and housing characteristics (similar to those collected during the Nairobi listing). The individual-girl level survey collected information on: socio-demographic characteristics, schooling history, educational attainment, social assets and networks, self-efficacy, locus-of-control, financial literacy, savings and livelihoods, marital and child-bearing aspirations, birth history, experience of physical and sexual harassment and violence, attitudes on FGM, self-reported health and nutrition, reproductive health knowledge, and HIV and AIDS risk perceptions. Girls also completed three tests that assessed literacy in the local language and English, mathematics (using excerpts from the Uwezo Kenya National Learning Assessment 2012) [44] and nonverbal cognition (using a subset of Raven’s Coloured Progressive Matrices) [45]. The survey was translated into Swahili and Somali, pilot-tested and revised based on feedback from interviewers prior to data collection and only women interviewers implemented the survey with the girls. The individual-girl level survey was only administered after written/assent consent were obtained both by the respondent’s parent/guardian and by the respondent herself.

### Analysis

At endline and at follow-up, the program evaluation will examine the average treatment effect across all the different intervention packages, while exploring the causal mechanisms underlying any impacts. This will be done using an intent-to-treat (ITT) analysis, comparing the average increase in the primary (and secondary) outcomes of girls in the baseline survey and each subsequent survey in one arm to the average increase in another arm. As per ITT, all girls in the original sample (and successfully re-interviewed later) who are randomized to study arms will remain part of the study regardless of noncompliance, protocol deviations, withdrawal from the intervention, or anything else that happens after randomization. ITT estimates are relevant for understanding the impact at the population level for a program like this in which it is likely that not all girls would agree to participate and some of those that do would be noncompliant. Primary analyses will be unadjusted, accounting only for clusters and strata in Wajir. In addition, adjusted analyses will be conducted controlling for baseline socio-demographic characteristics to address potential differences across arms at baseline or due to differential attrition, as well as to possibly increase precision. Propensity-score matching on baseline indicators will be used to select comparison girls from the external control site to minimize risk of selection bias when making comparisons with girls in the program sample.

### Ethical considerations

The study protocol was approved by the Population Council Institutional Review Board and the AMREF Ethical and Scientific Review Committee. In addition, the protocol was reviewed by the Kenyan National Commission for Science, Technology and Innovation to obtain research permits for study investigators.

## Discussion

The existing literature points to the potential value of investing in very young adolescents before critical negative life events – school dropout, unintended pregnancy, early marriage, and experience of sexual and gender based violence – have occurred. The evidence, however, is lacking on which combination of interventions would be the most impactful. Furthermore, in order to provide policy-relevant evidence that governments, donors, and other stakeholders can use to scale up successful interventions for girls, it is critical to understand the costs associated with each intervention and how much impact can be expected for the additional financial inputs.

At a 2012 international expert meeting hosted by the UK Department for International Development (DFID) and the Girl Hub, there was consensus that the field of girl-centred programming had made important advances in recent years but that there remained critical gaps about “What works, under what conditions, and for what girls-and how and why do these elements combine and impact each other?” [46]. The research findings from AGI-K are well positioned to make significant contributions to answering this question [47].

While the intervention packages are substantively the same across the two sub-studies (Nairobi and Wajir), the specifics of the interventions were designed to be culturally appropriate for each study area. Findings from both studies can be compared and conclusions made about whether the same packages of interventions had impacts on the same types of outcomes in both areas, and if not, why there were differences. Findings from Nairobi could be cautiously generalized to other urban slums in Kenya with similar populations and findings from Wajir cautiously generalized to other similar areas in Kenya, including semi-arid areas of Northeastern Kenya with similar populations. Findings that are similar across both of these two very different settings will be relevant for vulnerable girls throughout Kenya as well as elsewhere.

### Availability of data and materials

De-identified data will be made available in a publically available repository before submission of impact evaluation results for peer-reviewed publication.

## Additional file

Additional file 1: AGI-K baseline survey. (XLSX 2079 kb)

## Footnotes

1 In Nairobi, the implementing partner is Plan International; in Wajir, Save the Children.

2 In mid-2015 the official exchange rate was 98.5 KES to the U.S. dollar.

3 In 2013, Kenya began a process of decentralization (devolution), where the 8 provinces and their administrators were replaced by 47 counties led by governors and county commissioners, and districts were reorganized as sub-counties headed by deputy commissioners.

4 The program design initially included 10-year olds but this group was dropped from the sample as they were less likely to have had their first pregnancy within the four-year study period, so that their inclusion would have reduced the sample’s power.

5 For Kibera, because sampling was conducted based on a listing of households over a period of 3 months, the sample includes girls who were under 15 on November 20, 2015, and at least 11 on January 18, 2015.

6 Figures for Wajir do not take into account different number of observations in each cluster and therefore may understate the minimum detectable effect size.

7 The Kibera External Advisory Committee is a group including key stakeholders at the county level and convened by Plan International in Kibera for the purpose of providing general guidance and promoting research uptake. A parallel External Advisory Committee for Wajir County was convened by Save the Children.
